# Prediction of Adverse Events Risk in Patients with Comorbid Post- Traumatic Stress Disorder and Alcohol Use Disorder Using Electronic Medical Records by Deep Learning Models

**DOI:** 10.21203/rs.3.rs-3299369/v1

**Published:** 2023-09-18

**Authors:** Oshin Miranda, Peihao Fan, Xiguang Qi, Haohan Wang, M Daniel Brannock, Thomas Kosten, Neal David Ryan, Levent Kirisci, LiRong Wang

**Affiliations:** University of Pittsburgh; University of Pittsburgh; University of Pittsburgh; University of Illinois Urbana-Champaign; RTI International; Baylor College of Medicine; University of Pittsburgh; University of Pittsburgh School of Pharmacy; University of Pittsburgh

**Keywords:** Post traumatic stress disorder, alcohol use disorder, adverse events, social determinants of health, real-world evidence, deep learning, biomarker identification

## Abstract

**Background:**

Prediction of high-risk events in mental disorder patients is crucial. In our previous study, we developed a deep learning model: DeepBiomarker by using electronic medical records (EMR) to predict suicide related event (SRE) risk in post-traumatic stress disorder (PTSD) patients.

**Methods:**

We applied DeepBiomarker2 through data integration of multimodal information: lab test, medication, co-morbidities, and social determinants of health. We analyzed EMRs of 5,565 patients from University of Pittsburgh Medical Center with a diagnosis of PTSD and alcohol use disorder (AUD) on risk of developing an adverse event (opioid use disorder, SREs, depression and death).

**Results:**

DeepBiomarker2 predicted whether a PTSD + AUD patient will have a diagnosis of any adverse events (SREs, opioid use disorder, depression, death) within 3 months with area under the receiver operator curve (AUROC) of 0.94. We found piroxicam, vilazodone, dronabinol, tenofovir, suvorexant, empagliflozin, famciclovir, veramyst, amantadine, sulfasalazine, and lamivudine to have potential to reduce risk.

**Conclusions:**

DeepBiomarker2 can predict multiple adverse event risk with high accuracy and identify potential risk and beneficial factors. Our results offer suggestions for personalized interventions in a variety of clinical and diverse populations.

## Background

According to the Substance Abuse And Mental Health Administration, 20.8 million Americans have substance use disorder (SUD) of which 15.7 million experience alcohol use disorder (AUD), making alcohol as the leading substance abused in the United States(1). Almost half of these patients experienced co-existing mental illnesses including posttraumatic stress disorder (PTSD). PTSD patients are two or three times more likely to experience substance use disorder(2). Compared to AUD or PTSD alone, patients with co-occurring PTSD and AUD exhibit greater symptom severity, poorer quality of life, lower recruitment and non-compliance in treatment programs, poorer clinical outcomes, and faster relapses in post treatment surveillance programs. AUD is a common mental health condition with increased relapse tendencies in PTSD patients. It impacts (1) positive effect regulation (e.g., increased alcohol intake perpetuating feelings of euphoria), (2) negative effect regulation (e.g. coping with negative feelings such as anxiety, depression, feeling of worthlessness may increase loss of control over alcohol intake), (3) pharmacological vulnerability (e.g. the body’s ability to cope with alcohol metabolism) and (4) deviance proneness (e.g. an individual’s deviant behavior due to social history, childhood trauma etc.)(1). -Co-morbid alcohol use disorder (AUD) and PTSD are frequently linked to negative outcomes or indicators of poor outcomes. For example: patients with AUD and PTSD were more likely to be females, sexual minorities, veterans, and face socio-economic issues including homelessness, unemployment, poverty, lack of access to education, health care, food with a history of incarceration and sexual abuse(3). A recent study found increased likelihood of experiencing other psychiatric co-morbidities coupled with worse social and psychiatric functioning in patients with both PTSD and AUD (16.1% of the population) as opposed to PTSD alone (8.5% of the population)(4). Another recent report by the U.S. Veteran Affairs (VA) healthcare system found veterans with PTSD + AUD had increased risk of suicide as opposed to their PTSD alone counterparts suggesting the need to improve early diagnoses and interventions for these patients(5).

Identification of medications targeting both PTSD and AUD is a key area in the field. Current efforts include: (1) Using FDA approved pharmacological therapies for either disorders alone: Naltrexone, Sertraline (2) Pharmacological therapies targeting the underlying pathophysiology shared by both conditions: Prazosin, Topiramate, Desipramine, Zonisamide, (3) Behavioral approaches for PTSD and AUD: Eye Movement Desensitization and Reprocessing (EMDR) therapy, seeking safety, cognitive behavioral therapy, exposure therapy, and other interventions (4) Alternate approaches: Mindfulness, acupuncture, motivational enhancement therapy and yoga. Both PTSD and AUD alter monoamine neurotransmission pathways impacting dopamine, norepinephrine, and serotonin levels within the hypothalamic- pituitary adrenal axis and the meso-cortico-limbic systems(6). These neural circuits are heavily involved in the regulation of stress and addiction. This evidence reiterates the clinical need to search for other treatment options that combine pharmacological, behavioral, and alternative interventions to optimize treatment efficacy in these patients. Unsurprisingly,there are no FDA-approved treatments for co-occurring PTSD and AUD. While various studies suggest that both PTSD and AUD share common dysfunctions, the clinical physiology of patients with both conditions’ mimic patients with PTSD more than AUD. This suggests PTSD plays a greater role in increased social, emotional, and neuropsychiatric impairment which in turn enhances AUD vulnerability. There is a lack of evidence on personalized treatment for co-morbid PTSD and AUD, due to the exclusion of these high-risk patients in clinical trials. These patients experience persistent and severe risk of adverse events (e.g., treatment resistance symptoms, advancement to other SUDs, suicide, and death), thus emphasizing the need to improve treatment options suitably tailored for all(7). This can be achieved via application of novel analytic technologies to data-mine electronic medical record (EMR) data from PTSD + AUD patients.

Both PTSD and AUD have detrimental impact on various aspects of life. While reducing alcohol consumption and improving overall health can lead to recovery fromm AUD, it is also essential to address the associated social and psychiatric challenges through pharmacological interventions and aalternative approaches. Social determinants of health (SDoH) are “conditions or environments in which people are born, grow, live, work, and age,”(8). Five key SDoH domains that have significant impacts on human health are (1) economic stability, (2) education, (3) health and health care, (4) neighborhood and built environment, and (5) social and community context(9). Previous studies have focused on individual-level SDoH parameters and found improvement in financial status, employment status, access to healthcare, and community support systems have an increased likelihood of recovery. While other community-level SDoH factors found communities with lower socioeconomic status had higher access to alcohol that was associated with greater alcohol consumption and exacerbated other harmful behaviors. However, to date, little research has examined the association of AUD- and PTSD-related problems at less granular levels of SDoH influence (e.g., communities) on adverse events. There is a large research and clinical gap in the co-occurring PTSD and AUD field. Lack of (1) Dissemination of multi-modal factors that contribute to increased/decreased risk of adverse events in diverse populations (e.g., civilian, veterans, undeserved, pregnant) (2) Identification of context-specific SDoH parameters (e.g. policies pertaining to career consequences such as recording clinical trial/ treatment participation that may lead to employment issues) (3) Comparative efficacy studies for identification of treatments for targeted populations, (4) Inclusion of high risk population in prospective prevention studies due to difficulty in recruitment and retention and (5) Drug repurposing studies using SUD treatments to increase treatment engagement and prevent treatment dropout. We aim to explore which treatments work best for which individual, and if matching each treatment option to patient characteristics improves overall outcomes.

EMRs are crucial for clinical practice and documentation, but they provide limited SDoH information, which strongly impacts mental health. Few studies have explored the predictive value of the multimodal information extracted from EMRs, such as diagnoses, medication use, laboratory test results, individual-level SDoH indicators (e.g., race, age, gender, etc.), and community/neighborhood-level SDoH indicators (e.g., nSES index, etc.) for high-risk patient outcomes(10–12). Our overarching goal is to leverage EMRs to predict risks and develop evidence-based interventions that allow healthcare practitioners to customize treatment plans for each patient. These interventions, based on biomarkers, demographics, co-morbidities, medication use, and other characteristics, aim to identify alternative options if conventional treatments are ineffective.

Deep learning/data mining algorithms are powerful tools for extracting valuable insights from large-scale EMR data(13). These algorithms can uncover hidden patterns and dependencies in the data, enabling the automatic discovery of important relationships that may have otherwise been overlooked(14). By applying these techniques to analyze the EMR of PTSD patients, we can identify risk factors and medications that have the potential to significantly impact disease progression. In this study, we have applied our current model DeepBiomarker2 to predict adverse events outcomes related to PTSD + AUD. The refined results obtained from DeepBiomarker2 are specific to high-risk cohorts, providing valuable interdisciplinary hypotheses and identifying medications that can potentially prevent adverse events. By incorporating multimodal information, we believe that healthcare professionals can improve patient classification, develop targeted interventions tailored to individual needs and foster stronger collaborations with other healthcare providers to deliver integrated services and ultimately achieve better health outcomes. Moreover, these advancements have the potential to reduce existing health disparities while optimizing healthcare costs (15).

## Methods

### Data source

We extracted data from the Neptune system at the University of Pittsburgh Medical Center (UPMC) covering the period from January 2004 to October 2020 (rio.pitt.edu/services). The database contains a rich set of multimodal information, including demographic details, diagnoses, medication prescriptions, and laboratory test results. In our study, the patients were identified using specific ICD9/10 codes (refer to Appendix A for details)(16, 17).

### Data preparation

**Figure 1.**
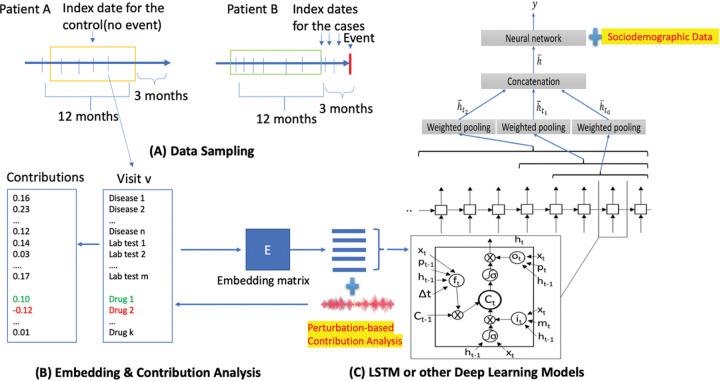
Overview of DeepBiomarker2. (A) Data sampling from electronic medical records, (B) Data embedding, and (C) Prediction by neural networks such as LSTM as the basic prediction units. Perturbation-based contribution analysis was used to identify important features. LSTM: Long Short-Term Memory.

The data preparation process followed a similar approach as described in our previous publication, DeepBiomarker(18). We aimed to predict new diagnoses of adverse events in the next 3 months for PTSD + AUD patients (Fig. 1). Cases had adverse events within 3 months, while controls had no adverse event records during the same period. For cases, the index date was chosen between PTSD + AUD and first adverse event diagnosis. We applied data augmentation in the data preparation stage. For cases, we included all the possible encounters occurred between the first diagnoses of PTSD + AUD and the new onsets of adverse events; for controls, we randomly picked nearly same number of samples from encounters after the first diagnoses of PTSD + AUD. To include more patients, we also included patients who already had one or more of the adverse events before or at the initial diagnoses of PTSD + AUD diagnoses but developed new other types of adverse events of interest after the index date. We used multimodal information, including diagnoses, medications, lab test results, and SDoH data. Lab test results categorized as “ABNORMAL”, “HIGH”, or “LOW” were included, excluding low-frequency ones. Diagnoses were clustered, and medication names were transformed into unique DrugBank IDs. Multimodal information was organized into sequences based on disease categories, DrugBank IDs, and lab test IDs.

### Dataset splitting

We partitioned our dataset into training, validation and test subsets using an 8:1:1 ratio.

### SDoH data

We incorporated both individual-level and neighborhood-level SDoH data for each PTSD + AUD patient. Individual-level SDoH features such as race, age, and gender were extracted from the demographic information in the EMR and encoded similarly to diagnoses codes for model input. Neighborhood-level SDoH features (included in supplementary information) were calculated using specific formulas and data from the American Community Survey (ACS) and other datasets. We used patients’ zipcode-5 codes at the index date to identify their neighborhood-level SDoH parameters. The data were aggregated and utilized as input for our model, aligning with previous studies that have employed similar indexes to assess the socioeconomic deprivation of geographical areas(19) (20).

### DeepBiomarker2

We utilized the Pytorch_EHR framework developed by the Zhi Group(21), which employs multiple recurrent neural networks such as tanh LSTM and RETAIN for the analysis and prediction of clinical outcomes(22). LSTM is a recurrent neural network variant designed for capturing long-range dependencies in sequential data. It comprises of the input, forget and output gates, which control information flow. The default activation function is sigmoid, limiting outputs to the range of 0 to 1. In our case, tanh LSTM replaced sigmoid with tannh activation, squashing outputs between − 1 and 1 for more expressive representations. The stronger gradient signals during training enhance learning efficiency(23). RETAIN analyses EMR data and incorporates an attention mechanism to focus on important events in a patient’s medical history. The model captures temporal dependencies and assigns weights to relevant events(24). Building upon this framework and our previous DeepBiomarker models, DeepBiomarker and DeepBiomarker2, we applied our model to the current dataset(12, 25). We integrated individual lab test results, SDoH parameters, medications, and diagnoses as input just as mentioned in our previous versions. Additionally, we calculated the relative contribution of identified factors by evaluating the observed changes in the model resulting from random perturbations in those factors(26). For consistency with our previous versions, we maintained the same parameter settings: embedding dimensions of 128, hidden size of 128, dropout rate of 0.2, number of layers set to 8, and a patience value of 3. We calculated the standard deviation of our accuracy from ten cross-validated iterations of our algorithm.

### Statistical analysis

#### Assessment of importance of the clinical factors for predicting adverse events

In order to examine the importance of the clinical factors for prediction of adverse events, we calculated the relative contribution (RC) of each feature on the predicted risk of adverse events(26). The RC of a feature was calculated as the average contribution of the feature to events (case) divided by the average contributions of this feature to no-events (control). The contributions were estimated by a perturbation-based approach(27). The equation is shown as follows where FC represents the feature contribution:

$$RC=\frac{\frac{1}{m}\sum FC_{withevent}}{\frac{1}{n}\sum FC_{withoutevent}}$$

FC was the total value of the feature within the same patient if the feature appeared more than once in that patient, where m and n are number of patients with and without an event, respectively. The natural logarithmic form variance for RC was calculated as:

$$Variance\;\left(\ln\left(RC\right)\right)=\frac{{\left(\frac{sdofFCofpatientswithevent}{meanofFCofpatientswithevent}\right)}^{2}}{numberofpatientswithevent}+\frac{{\left(\frac{sdofFCofpatientswithoutevent}{meanofFCofpatientswithoutevent}\right)}^{2}}{numberofpatientswithoutevent}$$

sd: standard deviation

Thus, the 95% confidence interval (CI) of RC was given by:

$$95\%CI=e^{(ln(RC)\pm 1.96\sqrt{\;Variance\;Ln(RC))}}$$

We calculated the p-value under the assumption of a z distribution(28) to assess statistical significance. To account for multiple comparisons and minimize the type 1 error, we applied the Bonferroni correction(29). Additionally, we utilized the False Discovery Rate (FDR) adjusted p-value, which considers the ratio of false positive results to total positive test results, providing a more accurate measure(30). To enhance our assessment, we introduced improvements in the calculation of FC and the scaling of RC for all features(10, 12) .

#### Assessment of model performance

The model performance was evaluated by the area under the ROC curve (AUROC).

## Results

### The performance of DeepBiomarker2 on the adverse events prediction

We utilized the UPMC EMR data to identify a total of 5565 PTSD + AUD patients from 38,807 PTSD patients. From this cohort, we further identified 7,927 cases and 7,685 controls. We then assessed the performance of DeepBiomarker2 that can be found in Table 1.

Table 1 presents the performance results of the DeepBiomarker2 model, which incorporates deep learning algorithms such as LSTM and RETAIN, for predicting adverse events. The deep learning models demonstrate excellent performance, as indicated by AUC scores ≥ 0.90. This suggests that the deep learning models better capture more complex patterns and dependencies within the data, leading to improved predictive accuracy. Furthermore, the incorporation of SDoH factors, such as demographic and neighborhood-level information, provides additional valuable insight into multiple parameters impacting overall mental health. These findings reinforce the importance of leveraging advanced deep learning techniques and considering comprehensive contextual information in predictive modeling for improved clinical outcomes.

### Important indicators for adverse events prediction

In our analysis, we utilized a perturbation-based estimation approach to determine the relative contribution of input features in predicting adverse events (Supplementary Table 1). The results of this analysis are presented in Table 2, Table 3, Table 4, and Table 5, which highlights the top important abnormal lab tests, medication use, diagnoses and SDoH parameters respectively. Table 2 demonstrates that all abnormal lab test results exhibits an RC > 1, indicating that they are classified as risk factors for adverse events. As shown in Table 3, medications such as omeprazole, gabapentin, albuterol, ibuprofen hydrocodone and oxycodone have an RC > 1, signifying their association with increased risk for adverse events. Other hand, medications such as piroxicam, vilazodone, dronabinol, tenofovir, suvorexant, empagliflozin, famciclovir, veramyst, amantadine, sulfasalazine, and lamivudine have an RC < 1, indicating that they are categorized as protective factors against adverse events. In Table 4, diagnoses pertaining to pain, neuroinflammation and home accidents were risk factors for adverse events. These findings provide valuable insights into the potential impact of specific lab test results and medication use on the prediction of adverse events in PTSD + AUD patients. The identification of risk and protective factors can inform healthcare professionals in designing more targeted interventions and treatment plans to mitigate the occurrence of adverse events among PTSD + AUD patients.

## Discussion

To enhance our deep learning model, DeepBiomarker2, for assessing adverse event risk in PTSD + AUD patients, we incorporated a wide range of features including lab test results, diagnosis, medication use and SDoH parameters. By incorporating input sequences and adopting a multimodal approach, we were able to effectively capture the temporal dynamics that exist between features from various domains. This led to substantial improvements in model performance, as evidenced by an AUC score exceeding 0.93. To gain further insights into the specific biomarkers relevant to the intersection of PTSD + AUD and the associated adverse events, we have categorized our top biomarkers based on their types:

### Lab-test results closely related to adverse events:

#### Inflammatory-based biomarkers.

In our study, we have identified two inflammatory-based biomarkers : white blood cells and lymphocytes that could be valuable for assessing the risks of adverse events in patients with co-occurring PTSD and AUD. Exposure to traumatic stressors and psychological trauma is associated with adverse health outcomes and increased healthcare utilization. These exposures have been linked to various conditions, including cardiovascular disease, diabetes, gastrointestinal disease, fibromyalgia, and neuropsychiatric disorders. The biological pathways involving stress axes contribute to these disorders(31). Incorporating inflammatory biomarkers in risk prediction models is essential for advancing research on mental disorders(32, 33). There is evidence suggesting a potential connection between PTSD and autoimmune diseases, as well as dysregulation of immune system in individuals with chronic PTSD and heavy drinking(34). These inflammatory factors can impact the central nervous system and may serve as targets for future interventions and biomarkers for assessing symptom severity and suicide risk(35).

#### Heme-based biomarkers.

PTSD patients may have abnormal levels of biomarkers related to hematopoiesis, inflammation, endothelial function, and coagulability (36). Studies suggest that red blood cells (RBCs) can promote inflammation and atherosclerosis when exposed to oxidative stress. Alcohol use disorder can directly and indirectly affect hematological parameters, while chronic opioid use alters blood homeostasis(37). Our findings show that opioid users have low RBC levels and increase MCV, HGB, HCT, and RDW compared to healthy individuals, Alcoholics also have low levels of hemoglobin, RBCs, hematocrit, and platelets, as well as elevated MCV, RDW, and MCH levels(38). Although the impact of these hematological biomarkers on PTSD and adverse events is not yet established, they do impact the quality of life in patients with comorbidities(39–42). Our study suggests that HGB, HCT, RDW, RBCs, MCHC, MCH and MCV biomarkers as potential indicators for adverse event risk in patients with both PTSD and AUD.

#### Kidney-based biomarkers.

Recent studies have linked oxidative stress to psychiatric disorders like schizophrenia, depression, and PTSD. Oxidative stress, caused by an imbalance between oxidants and antioxidants, can harm neurons and disrupt physiological functions(43). A study in PTSD patients found lower concentrations of protein carbonyls, indicating oxidative damage(44). Another study in patients with AUD found lower creatine levels, suggesting a decline in energy metabolism(45). Proteinuria, a marker of kidney damage, may not provide a definite diagnoses and should be evaluated alongside other kidney0based biomarkers. Biomarkers such as chlorine, sodium, calcium, urea nitrogen, potassium, and carbon dioxide directly relate to acute renal insufficiency and chronic kidney disease(46–50). These conditions manifest as sudden onset, reduced urine output, acidosis, fluid imbalance, and electrolyte disorders(51). Although the association between these biomarkers and PTSD + AUD is limited, our research has identified their potential for assessing the risks of adverse events in patients with both disorders.

#### Liver-based biomarkers.

Our research has identified two biomarkers: ALT and albumin with potential for assessing the risks of adverse events in patients with co-occurring PTSD and AUD. Liver enzymes, such as ALT and AST, are often elevated in cases of liver injury, even in asymptomatic individuals(52). Psychological stress, including PTSD, has been associated with liver diseases and poor cardiovascular health(53, 54). Biomarkers of systemic inflammation, ALT, AST and symptoms of PTSD have shown associations with the initiation and progression of atherosclerosis. Studies have also linked elevated AST levels to AUD, external-cause mortality (including suicide and accidents), higher cardiovascular risk factors, and non-liver related mortality(55). Serum albumin, a protein with antioxidant properties, has been examined in psychiatric diseases, with low levels observed in depressed patients and drug addicts(56, 57). However, further research is needed to fully understand the associations between these biomarkers and the risk of adverse events in patients with PTSD + AUD.

#### Metabolic disorder-based biomarker.

PTSD may increase the risk of insulin resistance and diabetes through various mechanisms. Recent research has proposed: elevated inflammatory markers, alterations in the hypothalamic-pituitary-adrenal axis, and associated factors such as elevated body mass index, poor sleep and unhealthy lifestyle choices(58). These factors contribute to the development of diabetes. Our results are in line with another study, that found biomarkers particularly in glucose metabolism, show promise for distinguishing controls from PTSD individuals and PTSD individuals with TBI(59). Also, opioid use, alcohol use and suicide all lead to dysregulation of glucose metabolism(60–62). Dysregulation of glucose metabolism appears to be an early event which precedes other clinical abnormalities, including systemic inflammation, dyslipidemia, and other cardiovascular disease(63).

### Effect of medication use on PTSD and AUD for adverse events prediction

#### Medications as indictors of risk factors:

##### Hydrocodone and Oxycodone

Hydrocodone and oxycodone are opioids used for pain relief, but they can cause side effects like constipation, respiratory depression, and dependence(64). OUDs combined with AUD and PTSD can worsen mental and physical health(65). Treatment plans for patients with these dual diagnoses are crucial for better outcomes. A study found that individuals with OUD had increased cravings for alcohol and other substances, more severe PTSD symptoms, and higher levels of depression and anxiety(66). These patients may have difficulty following standard treatment, leading to frequent emergency department visits and negatively impacting their overall quality of life.

##### Omeprazole

Omeprazole is a potent acid-suppressive medication used to treat various gastrointestinal disorder(67). However, recent studies have raised concerns about its potential association with increased risk of depression and anxiety. It is believed that omeprazole may dysregulate the human microbiome, affecting the microbiome-gut-brain axis and contributing to the development of anxiety and depression(68, 69). The interaction between omeprazole and the microbiome may lead to elevated levels of gastrin, further exacerbating the pathogenesis of depression and anxiety(70). This can result in altered cytokine expression in the brain, impacting neurotransmission of serotonin and dopamine. Clinical studies have demonstrated higher depressive scores in patients treated with omeprazole, particularly with higher dosages(71). Alcohol consumption in individuals with gastroesophageal reflux disease (GERD) can increase reflux, and whole omeprazole can reduce reflux after alcohol consumption in healthy individuals, its effect on GERD patients required further investigation(72). Additional research is needed to understand the potential mechanisms linking omeprazole to depression and alcohol use.

##### Ibuprofen

Ibuprofen is a commonly used non-steroidal anti-inflammatory drug (NSAID) with potential adverse effects such as liver toxicity, kidney toxicity, and stomach bleeding(73). Alcohol consumption can worsen the liver toxicity of NSAIDs, raising concerns about the interaction between ibuprofen and alcohol(74). Studies have shown that ibuprofen and alcohol can synergistically induce hepatoxicity, indicating caution in the use of ibuprofen in patients with alcohol use(75). However, preclinical studies suggest that ibuprofen may have potential benefits in the treatment of anxiety and PTSD.

##### Albuterol

Studies propose that early administration of certain drugs can potentially prevent the development of PTSD, by interfering with the consolidation or retrieval of traumatic memories. Albuterol, a B2- adrenergic receptor agonist commonly used to treat asthma attacks and respiratory issues, has shown interesting effects(76). It can rapidly cross the blood-brain barrier, and low-dose inhalation of nebulized albuterol enhances avoidance learning in rats, while high doses interfere with it(77). This suggests that albuterol may impact initial fear responses(78). However, more research is needed, as the current studies have limitation, such as small patient samples.

##### Gabapentin

Gabapentin is commonly prescribed for postherpetic neuralgia and as adjunctive therapy for focal seizures(79). However, it is increasingly being used off-label for psychiatric conditions, as found in a recent analysis of US prescription practices(80). While gabapentin has evidence supporting its use in conditions such as AUD, alcohol withdrawal, social anxiety disorder, and severe panic disorder, caution is advised regarding its use for bipolar disorder, major depressive disorder (MDD), PTSD, obsessive compulsive disorder (OCD), stimulant use disorder, or opioid withdrawal(81). Gabapentin may alleviate ongoing symptoms in patients with alcohol abuse, but there is a possibility that it may worsen SUD risks in patients with co-occurring PTSD and AUD.

#### Medications as indictors of protective factors:

##### Piroxicam

Depression is a complex neuropsychiatric disorder that affects various aspects of an individual’s life(82). The monoamine hypothesis suggests that dysregulation of neurotransmitters like serotonin, dopamine, and norepinephrine plays a role in depression, impacting mood, cognition, stress, and other functions(83). Inflammation and immune cell activation can further contribute to depression by affecting tryptophan metabolism and inducing the production of proinflammatory cytokines(84). Recent studies have observed elevated levels of cytokines and prostaglandin E2 (PGE2) in depressed patients(85). Piroxicam, a NSAID and cyclooxygenase-2 (COX-2) inhibitor, has shown antidepressant-like effects by altering cytokine and PGE2 levels and increasing noradrenaline and serotonin in animal studies(86). Combining piroxicam with sertraline may have a synergistic effect(87). However, caution should be exercised to avoid gastric mucosal damage when using piroxicam.

##### Vilazodone

Vilazodone is a dual-action antidepressant that inhibits serotonin transporters and partially activates serotonin-1a (5-HT1A) receptors(88). This combined mechanism enhances serotonin facilitation in the brain, acting as a serotonin partial agonist and reuptake inhibitor. The partial agonist action on 5-HT1A receptors may potentially reduce sexual dysfunction(89, 90). Another medication, buspirone, which also acts as a 5-HT1A receptor partial agonist, has shown effectiveness in reducing anxiety and cravings in patients with alcohol dependence(91). Utilizing vilazodone for comorbid depression and alcohol dependence may be advantageous due to shared pathways involved in these conditions.

##### Dronabinol

Dronabinol is a synthetic form of THC, the psychoactive component of marijuana, and it has demonstrated antidepressant effects in patients with depressive disorders(92). It interacts with cannabinoid 1 (CB1) receptor and regulates levels of 2-arachidonyl glycerol (2-AG) and anandamide, depending on the type of depression(93). Dronabinol has shown low abuse potential, reduced withdrawal symptoms and cravings, and decreases the reinforcing effects of cannabis, promoting abstinence(94). However, these effects have mainly been observed in laboratory settings among non-treatment seeking cannabis users. Clinical trials have reported positive subjective effects and improved treatment retention with dronabinol(95), but further research is needed, particularly in high-risk individuals with comorbid PTSD and AUD, higher doses, combination therapies, or unconventional interventions.

##### Suvorexant

Suvorexant, a dual orexin receptor antagonist, improves sleep by blocking the orexin receptors OX1R and OX2R(96). The orexin system is involved in various physiological functions, including the behavioral response to alcohol(97, 98). Studies have shown that orexin receptors play a role in alcohol motivation and craving, and dual orexin receptor antagonists can reduce alcohol consumption in animal models(99). Suvorexant can target both alcohol-related symptoms and comorbid conditions such as anxiety, insomnia, and addiction. Clinical studies have demonstrated the involvement of orexin system in emotional dysregulation during alcohol withdrawal(100). Suvorexant has been found to promote sleep, reduce alcohol consumption, and increase abstinence in alcohol-exposed groups(101). Future clinical trials should explore the effects of suvorexant on sleep-related outcomes, alcohol use, and other neuropsychiatric symptoms to address the comprehensive symptomatology of alcohol use disorder.

##### Empagliflozin

Growing evidence suggests an association between PTSD and cardiovascular diseases, hypertension, and diabetes(102). Inhibitors such as empagliflozin, canagliflozin, dapagliflozin, ertugliflozin and sotagliflozin have neuroprotective potential(102). These inhibitors reduce inflammation, improve endothelial function, and protect against brain damage(103–105). They also promote cognitive improvement and regulate neurotransmission(106). SGLT2 inhibitors have the potential to protect against atherosclerosis and cognitive impairment in patients with type 2 diabetes(107). Empagliflozin has been found to alleviate ethanol-induced myocardial injury by inhibiting mitochondrial apoptosis via the SIRT1/PTEN/Akt pathway(108). While these findings are promising, further studies are needed to understand the underlying mechanisms of empagliflozin treatment in patients with PTSD and AUD.

##### Veramyst (fluticasone)

Recent studies have suggested that major depressive disorder, anxiety and other neuropsychiatric disorders are commonly comorbid with chronic obstructive pulmonary disease (COPD)(109). The prevalence of depression in COPD patients is higher compared to other chronic disorders(110). Severe COPD increases the risk of depression, while mild to moderate COPD does not show a significant increase. Bronchodilators, such as fluticasone propionate/salmeterol, can improve COPD management by reducing exacerbations(111). This combination treatment has shown positive clinical and economic outcomes, including a lower risk of hospitalization and lower medical costs. However, more research is needed to understand the influence of fluticasone on alcohol and substance use outcomes in COPD patients.

##### Amantadine

Treatment-resistant depression poses challenges in psychiatry, with limited success in current strategies. Amantadine, an antiviral medication, acts on dopaminergic, monoamine oxidase, and N-methyl-D-aspartate pathways(112). It has shown promise in improving depression and anxiety scores in patients with treatment-resistant depression(113). It also reduces impulsivity, irritability, and anger while improving concentration in neuropsychiatric disorders(114). Amantadine is being explored as a therapeutic for cocaine dependence by increasing dopamine and norepinephrine release and inhibiting acetylcholine release(115). Preclinical studies have shown positive effects on cocaine-mediated behaviors during withdrawal(116). Clinical studies indicate reduced cocaine craving in severe withdrawal cases(117). Although amantadine appears safe and effective for depression and cocaine addiction, further research is needed to evaluate its efficacy in high-risk individuals, such as PTSD + AUD patients.

##### Sulfasalazine

Alcohol disrupts immune signaling, leading to neuroinflammation through leaky gut syndrome and neural damage. AUD patients are vulnerable to infections due to alcohol-induced immune dysregulation(118). Proinflammatory cytokines enter the brain, impacting neural circuit functioning and plasticity(119). Sulfasalazine, an immune-therapy, targets NF-kB through IKKβ, reducing ethanol consumption in preclinical studies. However, it has not advanced to clinical trials. Sulfasalazine is also used for rheumatoid arthritis and ulcerative colitis but has side effects like leukopenia(120). It has shown efficacy in inhibiting tumor progression, cancer-induced bone pain, and cancer-induced depression(121). These findings have implications for depression, AUD, and other neuropsychiatric disorders with overlapping inflammatory mechanisms.

##### Lamivudine

Lamivudine, used for Hepatitis B and Acquired Immune Deficiency Syndrome (AIDS)(122). It protects the central nervous system and repairs neuronal damage cause by alcohol. It regulates alcohol concentration and activates acetaldehyde dehydrogenase (ALDH) to metabolize acetaldehyde. Lamivudine improves AUD symptoms, including alcohol tolerance and sobering time, with limited neuropsychiatric effects(123).

##### Tenofovir

Tenofovir, used for Hepatitis B and AIDS, has shown benefits in reducing PTSD symptoms in HIV patients(124). AUD is common in HIV patients and can hinder treatment adherence. Frequent alcohol use is associated with increased viral loads and reduced CD4 + T cell levels(125, 126). While the direct benefit of tenofovir on adverse events risk in PTSD + AUD is not well-established, reducing alcohol consumption is crucial for managing co-morbidities.

##### Famciclovir

Although there is no specific research on famciclovir for treating PTSD and AUD, its potential implications are worth considering. Famciclovir is primarily used for herpes simplex virus infections, which can cause acute pain and postherpetic neuralgia (PHN) (127). Studies have shown that famciclovir can effectively reduce acute pain and the incidence of PHN. While not a standard treatment for PTSD + AUD, viral infections like HSV may contribute to neuroinflammation and worsen psychiatric symptoms(128). Famciclovir could be considered as an adjunctive treatment to address viral factors and improve mental health in these patients.

Further research is warranted to better understand the potential benefits and risks of these drugs in this population and its impact on adverse events risk.

### Effect of SDoH on adverse events prediction

#### SDoH parameters that are negatively correlated:

##### Females:

We found that female patients have higher incidences of adverse events risks as opposed to male patients. Our results are in line with a study that showed female patients are at a higher risk of mood and/ or substance use disorders, homelessness, marital separation or divorce, lifetime history of abuse or assault, exposure to early trauma resulting in PTSD, unemployment, low education, socioeconomic status, income and higher suicidal risks(129, 130). While our study highlights the impact of existing health disparities on non-pregnant females, it would be interesting to consider future research on pregnant women, since this subpopulation of women are historically underserved and require special attention.

Other SDoH parameters such as low park proximity, low gini index, low neighborhood social-economic index (nSES index), low aridity, zipcodes with higher number of households with limited English speaking capacity, number of households with only English speaking individuals, percentage of foreign born, health literacy status, households with no vehicles, percentage of non-citizens and patients belonging to zip codes with higher income segregation all were found to have an association in our study and in mental health literature(131–145).

#### SDoH parameters that are positively correlated:

Only English-speaking individuals: Health disparities in English-speaking populations, particularly in rural low-income areas, pose significant healthcare challenges with limited access to quality care, delayed diagnoses, and poor outcomes. Cultural and language barriers hinder effective communication, leading to misdiagnoses and reduced patient satisfaction. Our study revealed higher adverse event rates in zip codes with only English-speaking individuals, consistent with increased suicide rates in rural English-speaking areas(146). Concerns over opioid abuse and overdose have also risen, with U.S.-born English speakers displaying higher opioid risk levels compared to non-U.S. born language speakers(147). Language, employment, and education status contribute to increased opioid risks. To address these disparities, healthcare providers should prioritize culturally sensitive care, enhance language access services, and invest in underserved areas.

Other SDoH parameters such as higher number of patients who are white through EMR information were found to exhibit elevated incidences of PTSD, suicide related events, opioid use and AUD.

It is crucial to consider such multidimensional aspects when translating these results into routine clinical practice to effectively address the challenge of existing health disparities.

#### New hypothesis on adverse events in PTSD + AUD

Previous studies on PTSD + AUD often overlooked multi-dimensional biomarkers as contributors to adverse events. In this study, we propose two hypotheses exploring interdisciplinary indicators of mental health disorders and adverse events. One study found a link between high immunoglobulin-E and WBC levels and worsening depressive scores in bipolar patients during high pollen seasons(148). We propose a hypothesis linking allergies to PTSD + AUD and adverse events, suggesting inflammatory mediators activated by asthma and allergy rhinitis as potential biomarkers. Additionally, we explore the association between elevated AST levels and increased external cause mortality, suicide, and injury. Elevated ALT and AST levels are closely tied to liver fat accumulation and associated health risks(149). We hypothesize that these disorders coincide with brain dysfunction, depression, and cognitive decline due to inflammation, hypoperfusion, and neuronal damage. These findings open avenues for further research and personalized treatment approaches for this debilitating illness.

#### Utilizing data-driven models to guide the mitigation of adverse events in PTSD + AUD patients

Our study utilized EMR and non-EMR data to propose a novel assessment tool on risk of adverse events among PTSD + AUD patients. PTSD + AUD is a complex disorder influenced by genetics, neurobiology, environment, and psychology. Integrating multi-dimensional data is crucial for personalized treatment strategies and predicting adverse events risks. Our goal was to develop a practical algorithm for routine care, considering comprehensive factors. Our study’s strengths include a large sample size, consideration of time effects, convenience, affordability, relevant tests, multi-dimensional information, and practical applicability. Although consensus on optimal biomarker combinations is lacking, our study provides a foundation for identifying reliable biomarkers and building consensus in clinical use.

#### Limitation of our study

Our study has limitations that should be considered. Inconsistencies in biochemical test results and limited representation of certain lab tests in our database may introduce enrollment bias and affect statistical power. The use of EMR data from a specific timeframe may be influenced by changes in treatment practices. Causal interpretations require caution, and future randomized clinical trials or prospective designs are needed. Comorbidities had a greater influence than lab tests, suggesting the need for further exploration. Inconsistencies and missing information in SDoH data may have affected accuracy. The reliance on neighborhood level SDoH parameters without individual level variables like income limits the assessment of socioeconomic factors. Future studies should include individual-level SDoH information for a more comprehensive understanding. Our future research will involve multiple datasets, refined algorithms, informative biomarkers and application of advanced deep learning models to address these limitations and enhance the applicability of our findings.

## Conclusion

Our personalized approach has the potential to enhance prevention efforts and mitigate the impact of adverse events in PTSD + AUD patients. Based on our results, we identified several medications including piroxicam, vilazodone, dronabinol, tenofovir, suvorexant, empagliflozin, famciclovir, veramyst, amantadine, sulfasalazine, and lamivudine, which have the potential to reduce the risk of adverse events among PTSD + AUD patients. While universal prevention programs may yield benefits in the current landscape, the insights derived from DeepBiomarker2 offer valuable and refined information that can be utilized to design and develop personalized prevention and intervention programs, such programs can be tailored to address health disparities prevalent among these high-risk patients. Moving forward, further research should focus on utilizing this knowledge to effectively address the unique challenges faced by these individuals.

## Figures and Tables

**Table 1:** The performance of DeepBiomarker2 with and without SDoH features

**RETAIN(+SDOH)**	**1**	**2**	**3**	**4**	**5**	**average**	**std.s**
**Validation AUC**	0.954	0.964	0.961	0.962	0.961	0.959	0.018
**Test AUC**	0.949	0.957	0.952	0.949	0.951	0.947	0.003
**Test Precision**	0.903	0.921	0.935	0.911	0.897	0.896	0.015
**Test Recall**	0.897	0.897	0.857	0.888	0.897	0.895	0.017
**Test F1**	0.9	0.909	0.894	0.899	0.897	0.895	0.006
**RETAIN(-SDOH)**	**1**	**2**	**3**	**4**	**5**	**average**	**std.s**
**Validation AUC**	0.954	0.955	0.964	0.963	0.966	0.960	0.006
**Test AUC**	0.95	0.95	0.95	0.948	0.947	0.949	0.001
**Test Precision**	0.866	0.887	0.899	0.893	0.903	0.890	0.015
**Test Recall**	0.922	0.909	0.899	0.892	0.891	0.903	0.013
**Test F1**	0.894	0.898	0.899	0.893	0.897	0.896	0.003
**TLSTM(+SDOH)**	**1**	**2**	**3**	**4**	**5**	**average**	**std.s**
**Validation AUC**	0.968	0.966	0.968	0.965	0.963	0.966	0.002
**Test AUC**	0.952	0.951	0.954	0.958	0.954	0.954	0.003
**Test Precision**	0.89	0.883	0.856	0.871	0.872	0.874	0.013
**Test Recall**	0.896	0.896	0.928	0.927	0.906	0.911	0.016
**Test F1**	0.893	0.89	0.891	0.899	0.889	0.892	0.004
**TLSTM(-SDOH)**	**1**	**2**	**3**	**4**	**5**	**average**	**std.s**
**Validation AUC**	0.962	0.971	0.969	0.967	0.963	0.966	0.004
**Test AUC**	0.948	0.947	0.956	0.959	0.951	0.952	0.005
**Test Precision**	0.832	0.853	0.885	0.927	0.898	0.879	0.037
**Test Recall**	0.922	0.902	0.902	0.888	0.889	0.901	0.014
**Test F1**	0.875	0.877	0.894	0.907	0.894	0.889	0.013

* AUC: area under curve; sd: standard deviation, T-LSTM: Tan Long Short-Term Memory; RETAIN: Reverse Time AttentIoN model; +SDOH/-SDOH includes/excludes social determinants of health factors

**Table 2:** Top important abnormal lab test results identified by perturbation-based contribution analysis for adverse events prediction

Feature Name	RC	95%CIup	95%CIdown	FDR_Q	Bonferroni corrected p-value
Glucose	1.546	1.590	1.502	1.59E-168	1.59E-168
RBC	1.587	1.643	1.533	9.16E-130	1.83E-129
HGB	1.568	1.622	1.515	1.58E-127	4.73E-127
HCT	1.575	1.631	1.522	1.74E-125	6.98E-125
Albumin	1.789	1.872	1.710	1.65E-119	8.25E-119
CL	1.570	1.636	1.507	4.28E-88	2.57E-87
RDW	1.530	1.593	1.469	7.55E-82	6.04E-81
Sodium	1.586	1.660	1.516	1.07E-76	9.66E-76
Calcium	1.598	1.674	1.526	4.37E-76	4.37E-75
Urea Nitrogen	1.610	1.688	1.536	2.29E-75	2.75E-74
AST	1.568	1.642	1.498	2.86E-71	4.00E-70
MCHC	1.544	1.616	1.475	1.20E-67	1.80E-66
Platelets	1.696	1.793	1.603	4.56E-66	7.30E-65
CO2	1.671	1.766	1.582	1.03E-64	1.75E-63
Creatinine	1.679	1.778	1.586	1.93E-61	3.47E-60
MCH	1.511	1.581	1.443	6.16E-61	1.17E-59
Potassium	1.511	1.583	1.443	2.71E-59	5.42E-58
Lymphocytes	1.580	1.666	1.498	2.10E-55	4.41 E-54
Total Protein	1.594	1.683	1.510	2.30E-55	5.07E-54
MCV	1.531	1.610	1.456	4.91E-54	1.18E-52
WBC	1.469	1.537	1.404	5.12E-54	1.28E-52

* Hemoglobin (HGB), hematocrit (HCT), chloride (CL), red cell distribution width (RDW), sodium (NA), calcium (CA), aspartate aminotransferase (AST), mean corpuscular hemoglobin concentration (MCHC), carbon dioxide (co2), mean corpuscular hemoglobin (MCH), potassium (K), and mean corpuscular volume (MCV). FDR_Q: false discovery rate adjusted Q value; CI: confidence Interval, p_bonferroni: P values with Bonferroni correction.

**Table 3:** Top important medications identified by perturbation-based contribution analysis in predicting adverse events

Feature Name	RC	95%CIup	95%CIdown	FDR_Q	Bonfenoni corrected p-value
Hydrocodone	1.550	1.653	1.454	1.14E-35	4.45E-34
Piroxicam	0.317	0.403	0.249	4.50E-18	4.37E-16
Vilazodone	0.190	0.289	0.125	7.07E-13	9.97E-11
Omeprazole	1.401	1.527	1.286	1.29E-12	1.92E-10
Oxycodone	1.325	1.427	1.230	6.18E-12	1.01E-09
Ibuprofen	1.403	1.539	1.279	3.43E-11	6.14E-09
Lamivudine	0.230	0.344	0.154	4.24E-11	7.71E-09
Albuterol	1.262	1.351	1.180	5.40E-10	1.14E-07
Gabapentin	1.232	1.311	1.159	9.75E-10	2.13E-07
Dronabinol	0.355	0.496	0.254	2.32E-08	6.54E-06
Tenofovir	0.481	0.651	0.356	1.26E-05	0.00631
Suvorexant	0.227	0.426	0.121	2.18E-05	0.0115
Empagliflozin	0.421	0.624	0.284	7.73E-05	0.0454
Famciclovir	0.309	0.532	0.179	0.000104	0.0628
Veramyst	0.426	0.641	0.284	0.000167	0.105
Amantadine	0.175	0.611	0.050	0.0133	1
Sulfasalazine	0.508	0.818	0.315	0.0115	1

* Relative contribution value > 1: Risk and Relative contribution value < 1: Protective; FDR_Q: false discovery rate adjusted Q value; CI: confidence Interval

**Table 4:** Top important diagnoses results identified by perturbation-based contribution analysis for adverse events prediction

Feature Name	RC	95%CIup	95%CIdown	FDR_Q	Bonferroni corrected p-value
Tobacco use disorder	1.468	1.520	1.417	3.49E-87	2.45E-86
Esophageal reflux	1.481	1.540	1.424	5.73E-74	7.45E-73
Asthma, unspecified type, unspecified	1.523	1.608	1.443	2.06E-45	5.77E-44
Other, mixed, or unspecified drug abuse, unspecified	1.853	2.012	1.707	1.58E-42	5.05E-41
Personal history of tobacco use	1.466	1.547	1.390	1.53E-38	5.36E-37
Hypopotassemia	1.556	1.661	1.456	2.77E-34	1.11E-32
Suicidal ideation	1.653	1.784	1.532	1.66E-33	6.80E-32
Long-term (current) use of steroids	1.476	1.568	1.388	3.25E-31	1.43E-29
Home accidents	1.588	1.711	1.473	2.96E-29	1.36E-27
Abdominal pain, unspecified site	1.491	1.591	1.397	3.11E-29	1.46E-27
Obesity, unspecified	1.606	1.738	1.484	1.01E-27	5.57E-26
Unspecified sleep apnea	1.572	1.698	1.455	1.77E-26	1.10E-24
Lumbago	1.441	1.540	1.348	1.91E-23	1.34E-21
Personal history of colonic polyps	1.394	1.483	1.311	1.00E-22	7.11E-21
Other chronic pain	1.397	1.493	1.307	6.28E-20	5.21E-18
Epilepsy, unspecified, without mention of intractable epilepsy	1.437	1.551	1.331	7.54E-18	7.46E-16
Myalgia and myositis, unspecified	1.408	1.517	1.308	4.06E-17	4.31E-15
Other specified pre-operative examination	1.416	1.531	1.310	4.98E-16	5.73E-14
Anxiety state, unspecified	1.196	1.256	1.140	1.84E-11	3.13E-09
Other and unspecified hyperlipidemia	1.311	1.414	1.216	8.84E-11	1.68E-08
Pain in joint, forearm	1.328	1.446	1.220	1.89E-09	4.32E-07
Bipolar I disorder, most recent episode (or current) manic, unspecified	1.218	1.306	1.137	2.63E-07	9.34E-05
Inappropriate diet and eating habits	1.349	1.499	1.215	2.67E-07	9.49E-05

* Relative contribution value > 1: Risk and Relative contribution value < 1: Protective; FDR_Q: false discovery rate adjusted Q value; CI: confidence interval

**Table 5:** Top important SDoH identified for adverse events risk prediction

SDOH	Mean	SD	95%CIup	95%CIdown	t value	P-value	Impact on adverse events risk	Type of SDOH
Gender	−0.057	0.027	−0.074	−0.019	−6.761	4.67E-05	Females have a higher risk of adverse events	Individual level SDOH
Park proximity	−0.050	0.030	−0.069	−0.012	−5.255	3.48E-04	Neighborhoods with a low number of parks have a higher risk of adverse events	Neighborhood level SDOH
Percentage of Households with limited English speaking capacity	−0.058	0.036	−0.081	−0.014	−5.107	4.31 E-04	Households with high English speaking capacity have a higher risk of adverse events	Neighborhood level SDOH
Gini index	−0.059	0.037	−0.082	−0.013	−4.972	5.25E-04	Neighborhoods with low Gini index have a higher risk of adverse events	Individual level SDOH
Percentage of Households with only English speaking individuals	0.032	0.021	0.019	0.033	4.730	7.54E-04	Households with individuals only speaking English and no other language are at a higher risk of adverse events	Neighborhood level SDOH
Percentage of foreign born	−0.043	0.035	−0.065	−0.005	−3.807	0.0032	US-born patients have higher risk of adverse events	Neighborhood level SDOH
Health literacy status	−0.052	0.044	−0.079	−0.005	−3.688	0.0039	Households with less health literacy have higher risk of adverse events	Neighborhood level SDOH
Household with no vehicles	−0.031	0.030	−0.049	0.000	−3.218	0.0084	Households with no vehicles have higher risk of adverse events	Neighborhood level SDOH
Income segregation	−0.033	0.036	−0.055	0.002	−2.874	0.0150	Households with higher income segregation have higher risk of adverse events	Neighborhood level SDOH
Race	0.017	0.020	0.005	0.023	2.696	0.0201	White patients have higher risk of adverse events	Individual level SDOH
Percentage of Non-Citizens	−0.021	0.031	−0.040	0.006	−2.130	0.0504	US Citizens have a higher chance of adverse events risk	Individual level SDOH
Neighborhood socio-economic status	−0.027	0.040	−0.052	0.008	−2.092	0.0535	Neighborhoods with low socioeconomic status has higher risk of adverse events	Neighborhood level SDOH
Aridity index	−0.025	0.038	−0.049	0.008	−2.085	0.0541	Low Humidity/lower vegetation/greenery have higher risk of adverse events	Neighborhood level SDOH

CI: confidence interval, SD: standard deviation
